# Expression Profiles of the Individual Genes Corresponding to the Genes Generated by Cytotoxicity Experiments with Bortezomib in Multiple Myeloma

**DOI:** 10.4274/tjh.2015.0145

**Published:** 2016-12-01

**Authors:** Mehdi Ghasemi, Semih Alpsoy, Seyhan Türk, Ümit Y. Malkan, Şükrü Atakan, İbrahim C. Haznedaroğlu, Gürsel Güneş, Mehmet Gündüz, Burak Yılmaz, Sezgin Etgül, Seda Aydın, Tuncay Aslan, Nilgün Sayınalp, Salih Aksu, Haluk Demiroğlu, Osman İ. Özcebe, Yahya Büyükaşık, Hakan Göker

**Affiliations:** 1 Sentegen Biotechnology, Ankara, Turkey; 2 Bilkent University Faculty of Science, Department of Molecular Biology and Genetics, Ankara, Turkey; 3 METU Graduate School of Informatics Institute, Health Informatics Program, Clinic of Bioinformatics, Ankara, Turkey; 4 Hacettepe University Faculty of Pharmacy, Department of Biochemistry, Ankara, Turkey; 5 Hacettepe University Faculty of Medicine, Department of Internal Medicine, Division of Hematology, Ankara, Turkey; 6 Atatürk Training and Research Hospital, Clinic of Hematology, Ankara, Turkey

**Keywords:** Myeloma and other plasma cell dyscrasias, Neoplasia, cytogenetics, gene therapy, Molecular hematology

## Abstract

**Objective:**

Multiple myeloma (MM) is currently incurable due to refractory disease relapse even under novel anti-myeloma treatment. In silico studies are effective for key decision making during clinicopathological battles against the chronic course of MM. The aim of this present in silico study was to identify individual genes whose expression profiles match that of the one generated by cytotoxicity experiments for bortezomib.

**Materials and Methods:**

We used an in silico literature mining approach to identify potential biomarkers by creating a summarized set of metadata derived from relevant information. The E-MTAB-783 dataset containing expression data from 789 cancer cell lines including 8 myeloma cell lines with drug screening data from the Wellcome Trust Sanger Institute database obtained from ArrayExpress was “Robust Multi-array analysis” normalized using GeneSpring v.12.5. Drug toxicity data were obtained from the Genomics of Drug Sensitivity in Cancer project. In order to identify individual genes whose expression profiles matched that of the one generated by cytotoxicity experiments for bortezomib, we used a linear regression-based approach, where we searched for statistically significant correlations between gene expression values and IC50 data. The intersections of the genes were identified in 8 cell lines and used for further analysis.

**Results:**

Our linear regression model identified 73 genes and some genes expression levels were found to very closely correlated with bortezomib IC50 values. When all 73 genes were used in a hierarchical cluster analysis, two major clusters of cells representing relatively sensitive and resistant cells could be identified. Pathway and molecular function analysis of all the significant genes was also investigated, as well as the genes involved in pathways.

**Conclusion:**

The findings of our present in silico study could be important not only for the understanding of the genomics of MM but also for the better arrangement of the targeted anti-myeloma therapies, such as bortezomib.

## Abstract (tr)

**Amaç:**

Multipl miyelom (MM) günümüzde uygulanan yeni MM tedavilerine rağmen, refrakter hastalığın relapsı nedeniyle kür edilemeyen bir hastalıktır. In silico çalışmalar, MM’nin kronik seyrine karşı verilen klinikopatolojik savaşta alınan kararlar açısından oldukça önemlidir. Buradaki in silico çalışmanın amacı, bortezomib için yapılmış sitotoksisite çalışmalarında ortaya çıkan genlerle eşleşen özgün genleri ortaya koymaktır.

**Gereç ve Yöntemler:**

Biz bu çalışmada, potansiyel biyobelirteçleri ortaya koymak için araştırma konusuna uygun bir şekilde türetilmiş özetleyici veri seti üreterek in silico literatür taraması gerçekleştirdik. “Wellcome Trust Sanger” enstitüsünün 8 miyelom hücre serisi de olmak üzere toplam 789 kanser hücre serisini ilaç tarama verileriyle beraber içeren E-MTAB-783 veri seti ArrayExpress’den elde edilip, GeneSpring v.12.5 kullanılarak “Robust Multi-array analysis” normalize edildi. İlaç toksisite verisi “Genomics of Drug Sensitivity in Cancer” projesinden elde edildi. Biz bu çalışmada, eşleşen genleri saptamak amacıyla, gen ekspresyon değerleri ve IC50 verileri arasındaki istatistiksel açıdan anlamlı korelasyonları lineer regresyon temelli yaklaşım uygulayarak araştırdık. Sekiz hücre serisinde gen kesişimi tespit edildi ve bu hücre serileri ileri analiz için kullanıldı.

**Bulgular:**

Kullandığımız lineer regresyon modeli sayesinde 73 genin ve bazı gen ekspresyon düzeylerinin, bortezomibin IC50 değeri ile çok yakın korelasyon gösterdiğini tespit ettik. Tüm 73 geni hiyerarşik küme analizi ile incelediğimizde, iki ana kümede toplanan hücrelerin, görece duyarlı ve dirençli hücreler olduğunu gördük. Bütün önemli genlerin moleküler yolak ve fonksiyon analizi, yolaklara dahil olan genlerle beraber incelenmiştir.

**Sonuç:**

Gerçekleştirdiğimiz bu in silico çalışmada ortaya konan veriler, MM genomiğinin anlaşılması ve bortezomib gibi hedefe yönelik miyelom tedavilerinin daha iyi yönetilebilmesi açısından önemlidir.

## INTRODUCTION

Multiple myeloma (MM) is clinically, cytogenetically, and molecularly a very heterogeneous complicated neoplastic hematological disorder [1]. Numerous intra- and intercellular interactions, soluble/membrane-bound factors, and cell cycle machineries [2] represent potential targets of drug treatments in patients with MM [3]. Therefore, virtual drug treatments aimed at different targets can be explored using the computational models. Bortezomib is a targeted therapeutic drug for MM with high affinity, specificity, and selectivity for catalytic activity of proteasome. Bortezomib induces apoptosis in MM, inhibits the activation of nuclear factor-κB, suppresses survival of MM cells, and inhibits interleukin-6 triggered MM-cell proliferation, as well as inhibiting MM-cell adhesion in the bone marrow microenvironment [3,4,5,6,7]. Accurate preclinical predictions of the clinical efficacy of anti-MM drugs are needed.

MM is currently incurable due to refractory disease relapse even under novel anti-myeloma treatment [8]. Current challenges for the management of MM, including bortezomib drug treatment, are resistance development to drugs, increased unsustainable cost [9,10], lack of standardization in the therapeutic steps including stem cell transplantation, and morbidity and mortality due to drugs and/or ongoing resistant incurable neoplastic myeloma disease [4,5,11,12,13]. In silico studies are effective for key decision making during clinicopathological battles against the chronic course of MM [3,7,14,15]. The aim of this present in silico study is to identify individual genes whose expression profiles match that of the one generated by cytotoxicity experiments for bortezomib. Elucidation of the gene expression profiles (GEP) of the proteasome inhibitors in the pharmacobiological basis of MM is extremely important for the clinical activity of anti-MM drugs with regards to effectivity, safety, tolerability, toxicity, and pharmacoeconomy. The use of predictive simulation technology seems to be vital in designing therapeutics for targeting novel biological mechanisms using existing or novel chemistry [16].

## MATERIALS AND METHODS

### Public Expression and Drug Cytotoxicity Data

The myeloma cell line expression data were retrieved from ArrayExpress (E-MTAB-783) and consisted of transcriptomic profiles of 789 cancer cell lines from various types of cancer. Seven myeloma cell lines (ARH-77, IM-9, LP-1, L-363, OPM-2, RPMI-8226, and SK-MM-2) among the 789 cell lines were selected to be used in analyses after quality control. The drug cytotoxicity data of bortezomib, on the other hand, were retrieved from the Genomics of Drug Sensitivity in Cancer database of the Wellcome Trust Sanger Institute (http://www.cancerrxgene.org).

### Expression Data Preprocessing

GeneSpring software version 12.5 was used to extract raw data and background corrected gene expression data were generated. Further preprocessing was done using the Affy package for R and “Robust Multi-array analysis” normalization was applied to the data according to the Affy procedure.

### In Silico Classification of Myeloma Cell Lines and Identification of Candidate Gene Biomarkers

We used an in silico literature mining approach to identify potential biomarkers by creating a summarized set of metadata derived from relevant information [17,18,19]. To do that, a linear regression model was used to discover genes whose expression profiles correlated with bortezomib sensitivity as measured for 7 myeloma cell lines by IC50 values from the Genomics of Drug Sensitivity in Cancer database. All genes with a Pearson’s correlation coefficient related p-value below 0.01 and Pearson product-moment correlation coefficient value (r-value) higher than 0.9 were considered as candidate biomarker genes. Myeloma cell lines (SK-MM-2, OPM-2, U-266, RPMI-8226, ARH-77, L-363, IM-9, and LP-1) were hierarchically clustered based on determined biomarker genes, with Euclidian distance measures for both genes and arrays and complete linkage, using Cluster 3.0 software. In addition, we mapped these genes in biological pathways by using the Protein ANalysis THrough Evolutionary Relationships (PANTHER) classification system tool. The gene expression levels of cell lines were correlated with drug screening data (IC50 data) of bortezomib from the Wellcome Trust Sanger Institute Database. Drug toxicity data were obtained from the Genomics of Drug Sensitivity in Cancer project (http://www.cancerrxgene.org). 

## RESULTS

**Figure 1:**
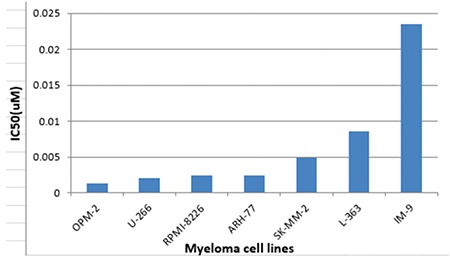
IC50 values for myeloma cell lines. As can be seen, the most resistant cell line to bortezomib is IM-9, while OPM-2 presents the most sensitive profile.

**Figure 2:**
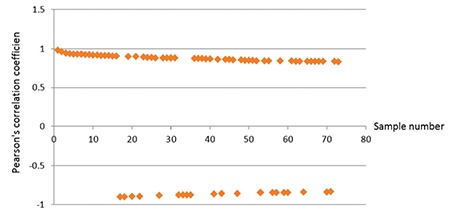
The correlation between gene expression and bortezomib IC50 values. Fifty-three genes are positively correlated with drug resistance while the rest show negative correlations.

**Figure 3:**
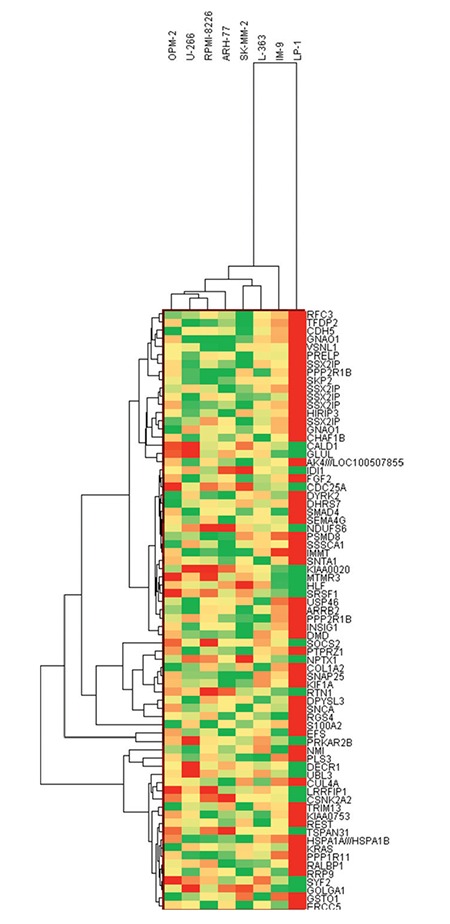
Clustering of multiple myeloma cell lines based on candidate gene biomarkers. Hierarchical clustering of myeloma cell lines according to 73 genes whose expressions show significant association with bortezomib chemosensitivity. Two major clusters are demonstrable, one containing relatively resistant cells and one containing less sensitive cells.

**Figure 4:**
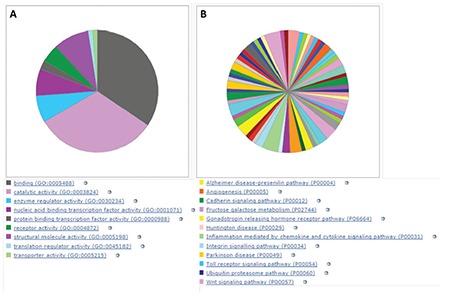
Biological pathway and molecular function analysis: A) biological pathway analysis of the genes whose expressions are correlated with bortezomib resistance in multiple myeloma cell lines; B) molecular function analyses of 73 genes that show a significant correlation with bortezomib resistance in multiple myeloma cell lines.

In order to identify individual genes whose expression profiles matched that of the one generated by cytotoxicity experiments for bortezomib, we used a linear regression-based approach, where we searched for statistically significant correlations between gene expression values and IC50 data [17,18,19]. The intersections of the genes were identified in 7 cell lines and used for further analysis. IC50 values of 7 MM cell lines after 72 h of treatment with bortezomib are shown in Figure 1. In this figure cells are sorted based on their sensitivity to bortezomib. Our linear regression model identified 73 genes. Genes with very good concordance between expression levels and bortezomib IC50 values are shown in Figure 2. When all 73 genes were used in a hierarchical cluster analysis, two major clusters of cells representing relatively sensitive and resistant cells could be identified, as seen in Figure 3. Pathway and molecular function analysis of all the significant genes is shown in Figure 4. Table 1 shows the genes involved in pathways. Table 1 also presents the families and subfamilies of these genes, suggesting that other members of these families might have effects on and responsibility for drug resistance. All of the proteins coded by these genes have key roles in cancer progression and some in metastasis.

## DISCUSSION

In this in silico study, the hierarchical clustering of myeloma cell lines according to bortezomib hemosensitivity biomarker genes has been described. The heat map represented the clustering of 8 myeloma cell lines based on 73 genes disclosing either bortezomib resistance or less sensitive myeloma cells. Likewise, the concordances between gene expression and IC50 values of 8 myeloma cell lines are shown for 73 genes. Furthermore, the relevant biological pathway analyses of the genes whose expressions are concordant with bortezomib cytotoxicity were explored via the molecular functional analyses of the 73 genes (Figures 2, 3, and 4). The genes involved in specific pathways regarding the proteasome inhibitors, and particularly bortezomib, are related to the critical pathological events of MM such as tumor angiogenesis and neoplastic signaling pathways (cadherin, integrin, Wnt, GnRH, ubiquitin), as well as chemokine-mediated inflammation (Table 1). Those pathways are essentially important in the biology of myeloma, such as the ubiquitin proteasome system, which plays a role in the regulation of most cellular pathways, and its deregulation in MM represents a target for proteasome inhibition via bortezomib [20]. Proliferation and apoptosis pathways are pathologically regulated by the ubiquitin-proteasome system, resulting in cellular neoplastic transformation in MM [21]. Targeting pathological angiogenesis in MM via bortezomib may delay tumor growth and reduce cytokine paracrine loops mediated by angiogenic factors [22]. Meanwhile, the signal transducers and activators of transcription proteins represent a family of cytoplasmic transcription factors that regulate a pleiotropic range of biological processes in MM [23]. Cell-cell interactions and cancer-initiating cells further complicate the biology of MM [24]. A previous study, in accordance with our present results, examined gene ontogeny related to bortezomib and suggested involvement in cellular development and carcinogenesis [25].

In the present study, by performing in silico correlation analysis, we determined genes whose expressions are correlated with bortezomib chemosensitivity in MM cell lines. Among 73 genes that are highly correlated with drug-resistant response (absolute Pearson r-value of >0.80), 20 genes showed a reverse correlation with chemosensitivity to bortezomib. This means that overexpression of these genes makes cancer cells more sensitive to bortezomib and the expressions of these genes are associated with good prognosis. Conversely, 53 genes are positively correlated to bortezomib response and make cells more resistant to drug treatment, and overexpression of these genes is associated with poor prognosis (supplementary data). We also tried to determine the pathways in which these genes are involved and figure out the relation between outcome and MM drug resistance profile by using another classification system. The PANTHER classification system was designed to classify proteins and their genes in order to facilitate high-throughput analysis. Proteins have been classified according to family and subfamily, molecular function, biological process, and pathway. Further in vitro and clinical validation studies are needed to determine and validate the exact role of each gene or panel of genes that are suggested in the present study as gene biomarkers for bortezomib-resistant response in MM cancer.

In 2007 Mulligan et al. assessed the feasibility of prospective pharmacogenomics research in multicenter international clinical trials of bortezomib in MM [26]. They tried to highlight those genes whose expressions are related to drug response and survival using bone marrow clinical samples by performing gene set enrichment analysis, analysis of clinical response, and overall survival analysis. The present study has two main differences from that study in terms of genomic approach and databases used. Our database came from established MM cell lines and the genomic approach was analysis of correlations between gene expression and drug response. Despite the two different approaches, we can see that many genes in this study and in that of Mulligan et al. overlap. On the other hand, our analysis shows some other genes that are able to predict response to bortezomib.

Cancer cell lines have a notable role in cancer drug discovery. Jaeger et al. found that drug sensitivity in cancer cell lines is not tissue-specific and recommended that, to get the most trustable results using cell lines, it will be necessary to include those cell lines’ molecular characteristics [27]. Similarly, in this experiment we did integrate those data into biological analysis, such as pathway analysis and hierarchical clustering.

The overall results of the present data mining study reveal the complicated nature of MM [28] and locate the drug bortezomib at the critical crossroads of the pathobiology of the disease, driving the clinical course of MM. For instance, the LP-1 cell line was found to be resistant to bortezomib in our present study (Figure 3). A previous study suggested that the expression of Apaf-1 might be predictive of the response to proteasome inhibition [29]. Based on our present results, patients with MM mimicking the molecular profile/behavior of LP-1 at any clinical evaluation point during the long-term clinical course of MM will be candidates for therapeutic regimens other than bortezomib. Ideally, those multiresistant MM patients should be single- or multiple-transplanted based on individual clinical responses [14]. We intend to test these hypotheses in future experiments designed to examine genomic profiles of the biological samples obtained from our MM patient cohort.

The results of our present study represent the rational basis for future molecular studies dealing with biological myeloma samples (peripheral blood and/or bone marrow) obtained from ‘real-life’ patients with MM. This issue is not just academically important since the proper selection of anti-myeloma drugs in everyday clinical practice during the long-term incurable advanced clinical forms of MM is challenging even to the most skilled clinicians. Randomized clinical trials (RCTs) usually compare drugs but do not decide on treatment strategies and proper selection of drug combinations [30], particularly for the handicapped myeloma patients with already present organ toxicities that are usually excluded from RCTs [12,13,31]. For real-life myeloma clinics, the pharmacobiological profile of the anti-myeloma drug together with the resistance profile [32] should be determined with the corresponding pathobiology of the MM disease course. This molecular approach could be particularly important for making decisions about hematopoietic stem cell transplantation for MM [14].

MM is a very heterogeneous disease [1]. Genetic changes could play a major role in prognosis in MM. However, in contrast to leukemias, no “good-risk” abnormalities have been described. Molecular analyses using GEP dissected the genetic basis of MM. Although various GEP-based signatures have been reported to identify high-risk myeloma disease and predict prognosis, the inability of GEP to predict clinical response in MM is also evident [1].

Barlogie et al., Shaughnessy et al., and Shaughnessy et al. showed the advantages of using GEP data to elucidate the molecular basis of resistance to chemotherapy as well as classification of MM patients in terms of poor prognosis and risk of relapse [33,34,35]. In this study, we determined those genes whose expressions are in correlation with bortezomib using GEP data.

Khin et al. generated patient-individualized estimations of initial response to chemotherapeutic agents in MM and time to relapse [36]. They designated an experimental platform with the specific intent of generating experimental parameters for a computational clinical model of personalized therapy in MM, while taking into consideration the limitations of working with patient primary cells and the need to incorporate elements of the myeloma tumor microenvironment. They suggested that myeloma patient-specific computational models, parameterized by in vitro platforms, could be combined with genomic datasets to better understand drug resistance in MM. Wang et al. developed a computational model of MM-bone marrow microenvironment interactions and clarified that intercellular signaling mechanisms implemented in this model appropriately drive MM disease progression [37]. Our findings in the present study also indicated that an understanding of the genomic myeloma dynamics might be useful for predictions of disease prognosis, as well as for proposing better therapeutic strategies for each patient with MM.

Bortezomib is able to induce tumor cell death by degradation of key proteins. It is employed as a first-line treatment in relapsed or resistant MM patients. However, bortezomib often induces a dose-limiting toxicity in the form of painful sensory neuropathy, which can mainly be reduced by subcutaneous administration or dose modification. Richardson et al. showed that some of the genes that are shown to related to bortezomib resistancy in the present study are also interestingly related to bortezomib-associated neurotoxicity [38]. It is suggested that those genes are involved in the pathways that control toxicity and resistancy [26,38].

The findings of our present in silico study could be important not only for the understanding of the genomics of MM but also for the better arrangement of targeted anti-myeloma therapies, such as bortezomib. Improvement in the understanding of MM pathogenesis will refine the molecular dissection of the disease, especially in the context of novel anti-myeloma drugs affecting the disease course. Genomics, proteomics, transcriptomics, and metabolomics studies (in silico, in vitro, in vivo) should be integrated to understand their significance in the management of MM, as well as to offer better therapeutics and treatment strategies to patients with MM.

## Ethics

Ethics Committee Approval: The research was performed in an in silico setting. Therefore, evaluation of the ethics committee was not required; Informed Consent: N/A. 

## Figures and Tables

**Table 1:**
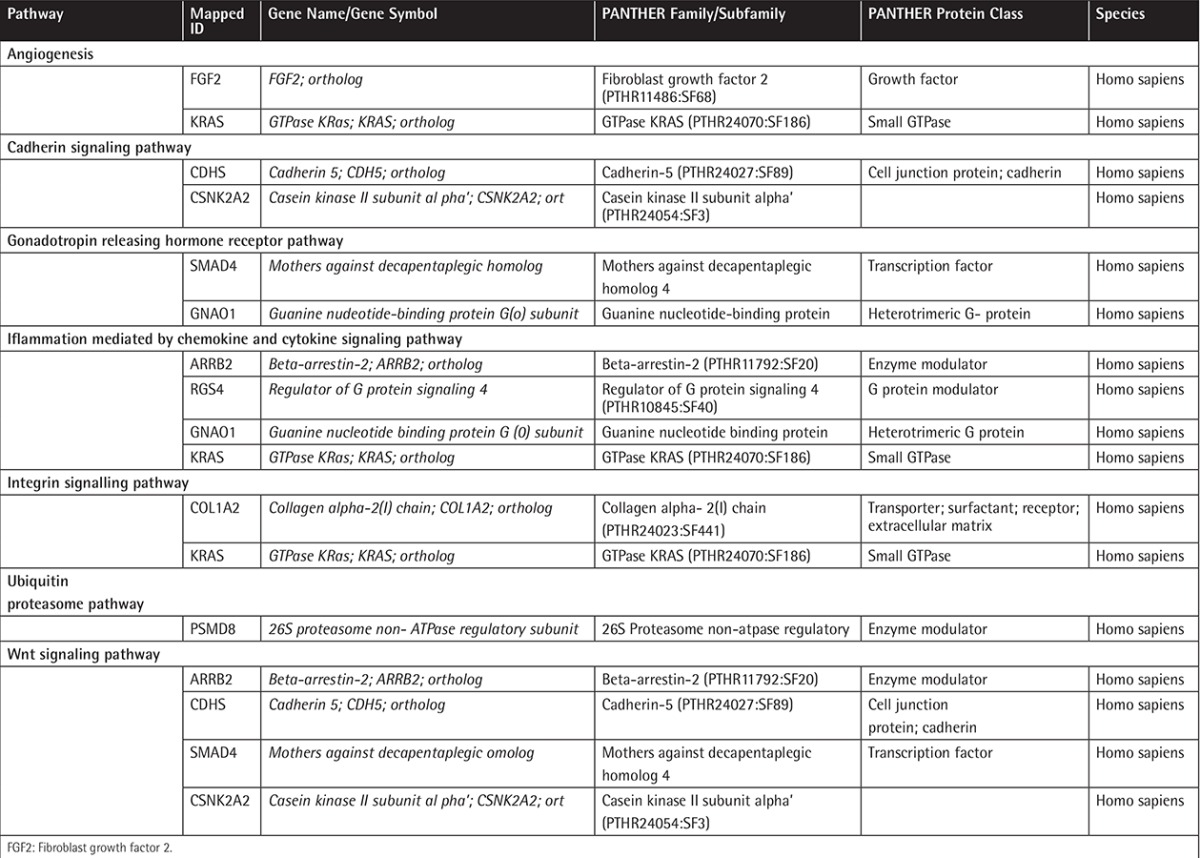
List of genes involved in specific pathways. The genes presented in this table are outcomes of Pearson correlation analysis done by using bortezomib chemosensitivity data and gene expression data for the multiple myeloma cell lines.
